# Pulmonary endarterectomy for chronic pulmonary thromboembolism, Timisoara department experience 2004-2012

**DOI:** 10.1186/1749-8090-8-S1-P149

**Published:** 2013-09-11

**Authors:** M Gaspar, W Klepetko, M Bogdan, P Deutsch

**Affiliations:** 1 Institute of Cardiovascular Disease, Timisoara, Romania; 2 Department of Thoracic Surgery, AKH, Vienna, Austria; 3 National Institute of Pneumology, Bucharest, Romania

## Background

The pulmonary thromboembolism, which may be subsequent or not to the deep venous thrombosis causes a serious disability, due to the severe pulmonary hypertension and right ventricular failure, with a life treating potential and very severe disability.

## Methods

In 2004-2012, we performed 26 PEA, 9 female and 17 males, age between 23 and 87 years old. The clinical suspicion of the diagnosis is confirmed by pulmonary artery angiography CT-with contrast. Surgical treatment, although marked with a high risk, represents the only chance in the advanced stages of the disease. The operation is performed in extracorporeal circulation, deep hypothermia 20*C, with several periods of total circulatory arrest, in order to facilitate the extraction of the thrombi from the pulmonary artery trunk and all the arterial pulmonary branches. As much as possible, all the thrombi should be extracted.

## Results

Two patients died after surgery, both with additional comorbidities and surgical procedures (anomalous venous drainage, aortic stenosis respectively). The patient selection and surgical technique are essential for early and late postoperative evolution. They are symptom-free, with very good exercise ability NYHA I, social reintegration, 8 years postoperative. Anticoagulation should remain as long.

## Conclusions

PEA is considered single curative treatment. Postoperative evolution in spite of longer stay on ICU is spectacular for the majority of the patients. They are only some centers doing this operation in Europe and the diagnosis is underestimated.

